# Capsular Switching and Other Large-Scale Recombination Events in Invasive Sequence Type 1 Group B *Streptococcus*

**DOI:** 10.3201//eid2211.152064

**Published:** 2016-11

**Authors:** Alefiya Neemuchwala, Sarah Teatero, Taryn B.T. Athey, Allison McGeer, Nahuel Fittipaldi

**Affiliations:** Public Health Ontario, Toronto, Ontario, Canada (A. Neemuchwala, S. Teatero, T.B.T. Athey, N. Fittipaldi);; Mount Sinai Hospital, Toronto (A. McGeer); University of Toronto, Toronto (A. McGeer, N. Fittipaldi)

**Keywords:** group B *Streptococcus*, capsular switching, recombination, whole-genome sequencing, bacteria, streptococci

## Abstract

We report several cases of recombination events leading to capsular switching among sequence type (ST) 1 group B *Streptococcus* strains. These strains otherwise shared a common genome backbone with serotype V ST1 strains. However, the genomes of ST1 serotype V strains and those of serotypes VI, VII, and VIII strains differed substantially.

Group B *Streptococcus* (GBS) is a major cause of invasive infections in neonates. GBS also causes invasive disease in adults, with incidence rates that have been increasing in North America, particularly among the elderly (*1*). A serologic reaction directed against the polysaccharide capsule permits the classification of GBS into 10 serotypes: Ia, Ib, and II–IX (*2*). Serotype V strains are most frequently associated with invasive infections in adults in North America (*3*,*4*). Multilocus sequence typing (MLST) permits differentiation of GBS strains into >700 sequence types (STs), which are grouped into a few clonal complexes (CCs) (*5*). A recent study found that 92% of serotype V GBS strains recovered from the blood of nonpregnant adults in Houston, Texas, USA, and Toronto, Ontario, Canada, belonged to ST1 (*4*).

Homologous recombination occurs frequently in GBS and can involve vast areas of the genome in some lineages (*6*,*7*). Recombination leading to capsular switching has been reported numerous times in GBS (*3*,*6*,*8*). Capsular switching might result from a single recombination event that replaces the *cps* locus encoding the capsular biosynthetic machinery, leaving the rest of the genome unchanged. However, it is also possible that receiving strains acquire a donor *cps* locus and additional genetic material from the same or other donor strain(s). Because multiple recombination events can endow the receiving GBS strain with an enhanced virulence arsenal, obtaining information about additional genome changes is important.

## The Study

We investigated 111 ST1 GBS isolates collected by the Toronto Invasive Bacterial Diseases Network during 2009–2015. This network is a population-based surveillance system for invasive bacterial diseases in metropolitan Toronto and Peel Region, Ontario, Canada (total population under surveillance ≈5.5 million) that includes all hospitals (n = 28) providing care to and all laboratories (n = 25) processing sterile site cultures from residents of the population area. MLST and serotyping were performed as previously described (*4*,*5*,*9*). Most (103) of these ST1 strains were serotype V, but 8 ST1 isolates were found to be serotype Ib, II, IV, VI, VII, or VIII (Table). These 8 strains represent an opportunity to investigate in more detail the molecular underpinnings of capsular switching in GBS. To this end, we sequenced the genomes of all 8 strains. We extracted genomic DNA by using the QIAGEN DNA minikit (QIAGEN, Toronto, Ontario, Canada) and prepared genomic libraries by using Nextera XT Kits (Illumina, San Diego, CA, USA). We sequenced libraries as paired-end reads with Illumina HiSeq (101 bp + 101 bp) or MiSeq (150 bp + 150 bp) instruments and deposited whole-genome sequencing data in the Sequence Read Archive of the National Center for Biotechnology Information (Table).

**Table:** Characteristics of 8 non–serotype V sequence type 1 group B *Streptococcus* isolates collected by the Toronto Invasive Bacterial Diseases Network, Ontario, Canada, 2009–2015*

Strain name	Serotype	Year isolated	Age group of patient	Source	SRA accession no.
NGBS217	Ib	2011	0–6 d	Blood	SRR3030375
NGBS748	II	2010	7–89 d	Blood	SRR3030378
NGBS814	II	2011	>60 y	Blood	SRR3030379
NGBS1098	IV	2012	7–89 d	Blood	SRR3030380
NGBS209	VI	2011	>60 y	Blood	SRR3030374
NGBS537	VI	2012	19–59 y	Blood	SRR3030376
NGBS015	VII	2009	>60 y	Blood	SRR3030373
NGBS621	VIII	2012	19–59 y	Blood	SRR3030377


*Sequence types as determined by multilocus sequence typing. SRA, Sequence Read Archive.

We next performed de novo genome assemblies using the A5 pipeline (*10*) and assessed the *cps* loci of the 8 strains. In all cases, blastn (http://blast.ncbi.nlm.nih.gov) comparisons identified the *cps* locus expected for the serotype identified by latex agglutination. To characterize recombination leading to capsular switching, we first used the variant ascertainment algorithm VAAL (*11*) to identify polymorphisms in each strain relative to the genome of serotype V ST1 strain SS1 (GenBank accession no. CP010867). The number of polymorphisms identified varied greatly between strains of the different serotypes. Strains of serotypes Ib, II, and IV had a relatively small number of polymorphisms, including single-nucleotide polymorphisms and small insertion/deletions, relative to the serotype V ST1 reference. The serotype Ib strain had 1,437 polymorphisms, the 2 serotype II strains had 1,115 and 816, respectively, whereas the serotype IV strain had 256. However, strains of the other 3 serotypes had a substantially higher number of polymorphisms relative to the genome of the serotype V ST1 strain SS1. The 2 serotype VI strains had 12,703 and 9,406 polymorphisms, respectively, the serotype VII strain had 4,117, and the serotype VIII strain had 3,471. By using custom scripts and the R software environment (https://www.r-project.org), we created plots of polymorphism distribution relative to the genome of the reference strain with a sliding window of 5 kbp. We observed a nonrandom polymorphism distribution in all 8 strains (Technical Appendix).

We next plotted the polymorphisms identified in the strains against the genome of serotype V reference strain SS1 by using BRIG (*12*) and assessed recombination by using BratNextGen (run with 20 iterations and 100 replicates at p = 0.05) (*13*). The analysis showed that serotype Ib, II, and IV strains had a genome background similar to the ST1 serotype V reference strain but that each had experienced horizontal transfer of genome sequences of differing sizes, all of which included the *cps* locus. Namely, serotype Ib strain NGBS217 had exchanged a DNA region of ≈200 kb and acquired a *cpsIb* locus (Figure, panel A). Serotype II strains NGBS814 and NGBS748 had exchanged DNA regions of ≈189 kb and ≈152 kb, respectively, and gained a *cpsII* locus (Figure, panel B). Recombination in the serotype IV strain NGBS1098 was less extensive (≈79 kb) but resulted in acquisition of a *cpsIV* locus (Figure, panel C). Thus, in strains of these 3 serotypes, capsular switching most likely resulted from a single recombination event that replaced the original *cpsV* locus of the receiving strains with those of the donor strains but left the rest of the genome of the receiving strains unchanged. We speculate that these recombination events most likely occurred by conjugation and that DNA exchange took place in the human gut or urogenital tract. These body sites can be colonized by multiserotype GBS populations (*14*).

**Figure:**
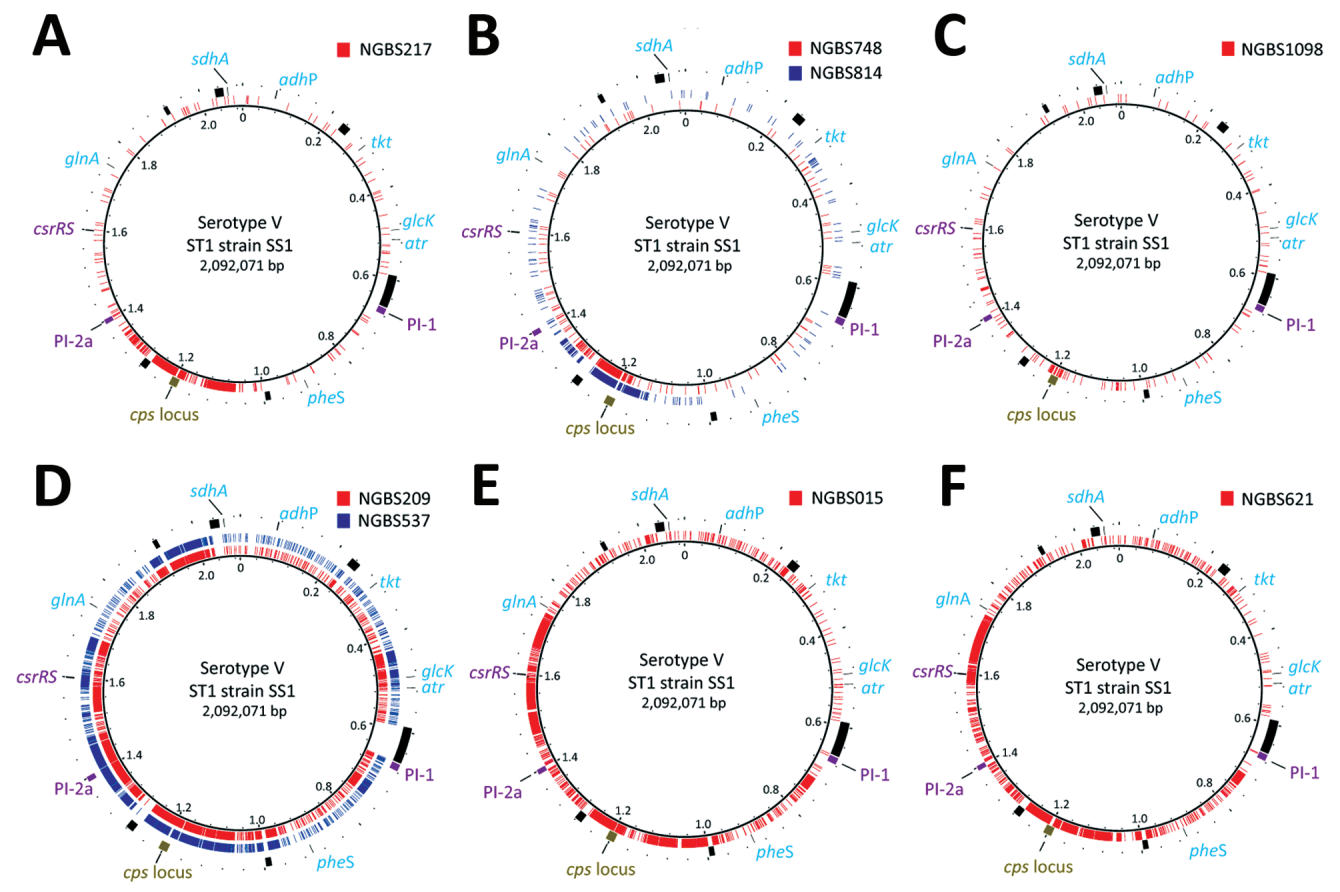
Extent of recombination among 8 non–serotype V sequence type (ST) 1 group B *Streptococcus* isolates collected by the Toronto Invasive Bacterial Diseases Network, Ontario, Canada, 2009–2015. A) Serotype Ib strain NGBS217. B) Serotype II strains NGBS748 and NGBS814. C) Serotype IV strain NGBS1098. D) Serotype VI strains NGBS209 and NGBS537. E) Serotype VII strain NGBS015. F) Serotype VIII strain NGBS621. Polymorphisms identified in non–serotype V ST1 strains (illustrated in different colors, with each line representing a polymorphism) are plotted against the genome sequence of reference serotype V ST1 strain SS1 (innermost circle). The *cps* locus is shown in gold in the outermost circle. Genome landmarks, such as genes used in the multilocus sequence typing scheme (*adhP, atr, tkt, glcK, sdhA, glnA,* and *pheS*) are shown in light blue. Mobile genetic elements of strain SS1 are depicted in black. Polymorphisms mapping to mobile genetic elements identified in the various non–serotype V ST1 strains were not included in the analysis. ST1, sequence type 1.

In contrast with those results, the genomes of strains of serotypes VI, VII, and VIII differed extensively from the reference serotype V strain. Indeed, recombination detected by BratNextGen involved >50% of the genomes of these isolates, even though they were found to be ST1 by MLST (Figure, panels D–F). Based on the available data, it is difficult to ascertain if those strains were originally ST1 strains that acquired a *cps* locus and many other genomic regions by recombination in apparently multiple recombination events or, alternatively, if the isolates under investigation originally possessed the *cps* locus corresponding to their serotype and acquired by recombination from unknown ST1 donors the different portions of the genome that contain all genes used in the MLST scheme. In the latter scenario, and in particular for serotype VI strains, the increased number of polymorphisms in ST1-like areas of the genome suggests that the putative ST1 donor was genetically not closely related to the serotype V ST1 reference strain SS1. 

## Conclusions

Our data suggest that extensive recombination might be a key contributor to clonal diversification and emergence of GBS serotypes VI, VII, and VIII, which are less often identified in North America but have substantial regional dominance in other geographic areas (*15*). MLST is a useful approach to begin to examine the genetic relationships of GBS strains. In some cases, particularly when temporally and geographically related isolates are evaluated, this approach also permits initial prediction of recombination events (*3*,*5*–*8*). Whole-genome sequencing extends MLST capabilities and enables precise identification and characterization of genetic variation attributable to extensive recombination in this opportunistic pathogen. Although the polysaccharide-based, trivalent conjugate GBS vaccine under development offers great promise, frequent capsular switching suggests that subunit vaccines based on antigens expressed by all GBS serotypes might offer enhanced protection.

Technical Appendix Results of a sliding-window analysis of the distribution of single-nucleotide polymorphisms identified in the genomes of 8 non–serotype V sequence type 1 group B *Streptococcus* isolates collected by the Toronto Invasive Bacterial Diseases Network, 2009–2015, relative to the genome of serotype V sequence type 1 reference strain SS1.

## References

[1] Skoff TH, Farley MM, Petit S, Craig AS, Schaffner W, Gershman K, Increasing burden of invasive group B streptococcal disease in nonpregnant adults, 1990-2007. Clin Infect Dis. 2009;49:85–92. 10.1086/599369 19480572

[2] Lancefield RC. A serological differentiation of specific types of bovine hemolytic streptococci (group B). J Exp Med. 1934;59:441–58. 10.1084/jem.59.4.441 19870257 PMC2132330

[3] Teatero S, McGeer A, Low DE, Li A, Demczuk W, Martin I, Characterization of invasive group B *streptococcus* strains from the greater Toronto area, Canada. J Clin Microbiol. 2014;52:1441–7. 10.1128/JCM.03554-13 24554752 PMC3993709

[4] Flores AR. Galloway-Peña J, Sahasrabhojane P, Saldaña M, Yao H, Su X, et al. Sequence type 1 group B *Streptococcus*, an emerging cause of invasive disease in adults, evolves by small genetic changes. Proc Natl Acad Sci U S A. 2015;112:6431–6. 10.1073/pnas.1504725112 PMC4443349 25941374

[5] Jones N, Bohnsack JF, Takahashi S, Oliver KA, Chan MS, Kunst F, Multilocus sequence typing system for group B *streptococcus.* J Clin Microbiol. 2003;41:2530–6. 10.1128/JCM.41.6.2530-2536.2003 12791877 PMC156480

[6] Brochet M, Rusniok C. Couvé E, Dramsi S, Poyart C, Trieu-Cuot P, et al. Shaping a bacterial genome by large chromosomal replacements, the evolutionary history of *Streptococcus agalactiae.* Proc Natl Acad Sci U S A. 2008;105:15961–6. 10.1073/pnas.0803654105 PMC2572952 18832470

[7] Da Cunha V, Davies MR, Douarre PE, Rosinski-Chupin I, Margarit I, Spinali S, ; DEVANI Consortium. *Streptococcus agalactiae* clones infecting humans were selected and fixed through the extensive use of tetracycline. Nat Commun. 2014;5:4544. 10.1038/ncomms5544 25088811 PMC4538795

[8] Bellais S, Six A, Fouet A, Longo M, Dmytruk N, Glaser P, Capsular switching in group B *Streptococcu*s CC17 hypervirulent clone: a future challenge for polysaccharide vaccine development. J Infect Dis. 2012;206:1745–52. 10.1093/infdis/jis605 23002446

[9] Teatero S, McGeer A, Li A, Gomes J, Seah C, Demczuk W, Population structure and antimicrobial resistance of invasive serotype IV group B *Streptococcus*, Toronto, Ontario, Canada. Emerg Infect Dis. 2015;21:585–91. 10.3201/eid2014.140759 25811284 PMC4378482

[10] Tritt A, Eisen JA, Facciotti MT, Darling AE. An integrated pipeline for de novo assembly of microbial genomes. PLoS One. 2012;7:e42304. 10.1371/journal.pone.0042304 23028432 PMC3441570

[11] Nusbaum C, Ohsumi TK, Gomez J, Aquadro J, Victor TC, Warren RM, Sensitive, specific polymorphism discovery in bacteria using massively parallel sequencing. Nat Methods. 2009;6:67–9. 10.1038/nmeth.1286 19079253 PMC2613166

[12] Alikhan NF, Petty NK, Ben Zakour NL, Beatson SA. BLAST Ring Image Generator (BRIG): simple prokaryote genome comparisons. BMC Genomics. 2011;12:402. 10.1186/1471-2164-12-402 21824423 PMC3163573

[13] Marttinen P, Hanage WP, Croucher NJ, Connor TR, Harris SR, Bentley SD, Detection of recombination events in bacterial genomes from large population samples. Nucleic Acids Res. 2012;40:e6. 10.1093/nar/gkr928 22064866 PMC3245952

[14] Ferrieri P, Hillier SL, Krohn MA, Moore D, Paoletti LC, Flores AE. Characterization of vaginal & rectal colonization with multiple serotypes of group B streptococci using multiple colony picks. Indian J Med Res. 2004;119(Suppl):208–12. 15232197

[15] Lachenauer CS, Kasper DL, Shimada J, Ichiman Y, Ohtsuka H, Kaku M, Serotypes VI and VIII predominate among group B streptococci isolated from pregnant Japanese women. J Infect Dis. 1999;179:1030–3. 10.1086/314666 10068604

