# Supplementary material for: Capsular Switching and Other Large-Scale Recombination Events in Invasive Sequence Type 1 Group B Streptococcus
Source: Emerg Infect Dis. 2016 Nov;22(11):1941–4. doi: 10.3201//eid2211.152064 (PMC5088006; doi:10.3201//eid2211.152064)
Supplement: Technical Appendix — Results of a sliding-window analysis of the distribution of single-nucleotide polymorphisms identified in the genomes of 8 non–serotype V sequence type 1 group B Streptococcus isolates collected by the Toronto Invasive Bacterial Diseases Network, 2009–2015, relative to the genome of serotype V sequence type 1 reference strain SS1. [file 15-2064-Techapp-s1.pdf]

Article DOI: http://dx.doi.org/10.3201/eid2211.152064

Capsular Switching and Other Large-Scale Recombination Events in Invasive Sequence Type 1 Group B Streptococcus

Technical Appendix.

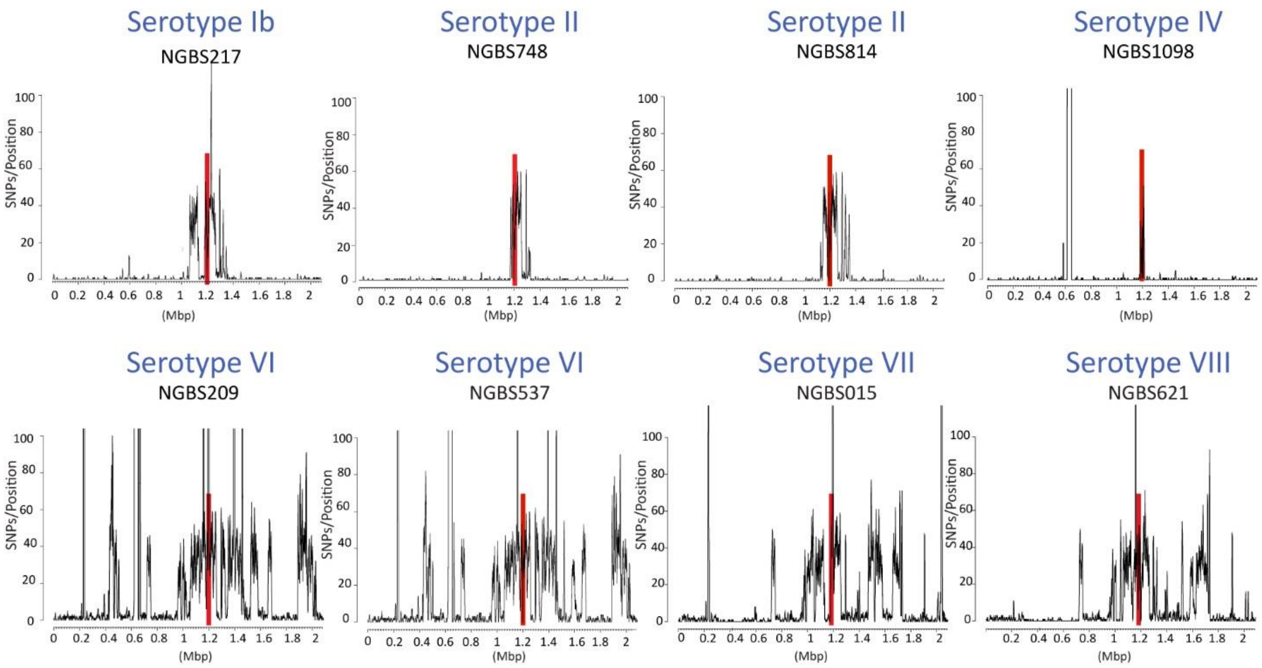

Figure. Results of a sliding-window analysis of the distribution of single-nucleotide polymorphisms identified in the genomes of 8 non–serotype V sequence type 1 group B *Streptococcus* isolates collected by the Toronto Invasive Bacterial Diseases Network, 2009–2015, relative to the genome of serotype V sequence type 1 reference strain SS1. The position of the *cps* locus in the genome of strain SS1 is highlighted in red. Polymorphism distribution was nonrandom in all strains. SNPs, single-nucleotide polymorphisms.
